# mTOR inhibitors reduce enteropathy, intestinal bleeding and colectomy rate in patients with juvenile polyposis of infancy with *PTEN-BMPR1A* deletion

**DOI:** 10.1093/hmg/ddab094

**Published:** 2021-04-02

**Authors:** Henry Taylor, Dilay Yerlioglu, Claudia Phen, Antje Ballauff, Natalia Nedelkopoulou, Isabel Spier, Inés Loverdos, Veronica B Busoni, Jürgen Heise, Peter Dale, Tim de Meij, Kevin Sweet, Marta C Cohen, Victor L Fox, Emmanuel Mas, Stefan Aretz, Charis Eng, Stephan Buderus, Mike Thomson, Isabel Rojas, Holm H Uhlig

**Affiliations:** Department of Surgery and Cancer, Imperial College London, London SW7 2BX, UK; Faculty of Medicine, Istanbul University, Istanbul, Fatih 34093, Turkey; Division of Pediatric Gastroenterology, Hepatology, and Nutrition, University of Texas Southwestern Medical Center, Dallas, TX 75390, USA; Zentrum für Kinder- und Jugendmedizin Gastroenterology, HELIOS Klinikum Krefeld, Krefeld, Nordrhein-Westfalen 47805, Germany; Pediatric Gastroenterology, Sheffield Children’s Hospital NHS Foundation Trust, Sheffield, Yorkshire S10 2TH, UK; Institute of Human Genetics, Medical Faculty, University of Bonn, Bonn, Nordrhein-Westfalen 53012, Germany; National Centre for Hereditary Tumor Syndromes, University Hospital Bonn, Bonn, Nordrhein-Westfalen 53126, Germany; Pediatric Gastroenterology Hepatology and Nutrition Unit, Parc Taulí Hospital Universitari, Institut d’Investigació i Innovació Parc Taulí I3PT, Universitat Autònoma de Barcelona, Sabadell, Catalonia comunidad 08208, Spain; Pediatric Gastroenterology, Hepatology and Liver-Intestine Transplantation Division, Hospital Italiano de Buenos Aires, Ciudad Autónoma de Buenos Aires, Argentina; Zentrum für Kinder- und Jugendmedizin Gastroenterology, HELIOS Klinikum Krefeld, Krefeld, Nordrhein-Westfalen 47805, Germany; Royal Gwent Hospital, Newport NP20 2UB, UK; VU University Medical Center, Amsterdam 1081, The Netherlands; Division of Human Genetics, Ohio State University Wexner Medical Center, Columbus, OH 43210, USA; Histopathology Department, Sheffield Children’s Hospital NHS Foundation Trust, Sheffield, Yorkshire S10 2TH, UK; Division of Gastroenterology, Hepatology and Nutrition, Boston Children’s Hospital, Boston, MA 02115, USA; Unité de Gastroentérologie, Hépatologie, Nutrition, Diabétologie et Maladies Héréditaires du Métabolisme, Hôpital des Enfants, CHU de Toulouse, and IRSD, Université de Toulouse, INSERM, INRA, ENVT, UPS, Toulouse, Occitanie 31300, France; Institute of Human Genetics, Medical Faculty, University of Bonn, Bonn, Nordrhein-Westfalen 53012, Germany; National Centre for Hereditary Tumor Syndromes, University Hospital Bonn, Bonn, Nordrhein-Westfalen 53126, Germany; Genomic Medicine Institute, Lerner Research Institute, and Taussig Cancer Institute, Cleveland Clinic, Cleveland, OH 44195, USA; Department of Genetics and Genome Sciences, and CASE Comprehensive Cancer Center, Case Western Reserve University School of Medicine, Cleveland, OH 44106, USA; GFO-Kliniken Bonn, St. Marien-Hospital, Bonn, Nordrhein-Westfalen 53115, Germany; Pediatric Gastroenterology, Sheffield Children’s Hospital NHS Foundation Trust, Sheffield, Yorkshire S10 2TH, UK; Division of Pediatric Gastroenterology, Hepatology, and Nutrition, University of Texas Southwestern Medical Center, Dallas, TX 75390, USA; Translational Gastroenterology Unit, University of Oxford, Oxford, Oxfordshire OX3 9DU, UK; Department of Pediatrics, University of Oxford, Oxford, Oxfordshire OX3 9DU, UK; Biomedical Research Centre, University of Oxford, Oxford, Oxfordshire OX4 2PG, UK

## Abstract

Ultra-rare genetic disorders can provide proof of concept for efficacy of targeted therapeutics and reveal pathogenic mechanisms relevant to more common conditions. Juvenile polyposis of infancy (JPI) is caused by microdeletions in chromosome 10 that result in haploinsufficiency of two tumor suppressor genes: phosphatase and tensin homolog deleted on chromosome 10 (*PTEN*) and bone morphogenetic protein receptor type IA (*BMPR1A*). Loss of *PTEN* and *BMPR1A* results in a much more severe phenotype than deletion of either gene alone, with infantile onset pan-enteric polyposis and a high mortality rate. No effective pharmacological therapy exists. A multi-center cohort analysis was performed to characterize phenotype and investigate the therapeutic effect of mammalian target of rapamycin (mTOR) inhibition (adverse events, disease progression, time to colectomy and mortality) in patients with JPI. Among 25 JPI patients identified (mean age of onset 13 months), seven received mTOR inhibitors (everolimus, *n* = 2; or sirolimus, *n* = 5). Treatment with an mTOR inhibitor reduced the risk of colectomy (hazard ratio = 0.27, 95% confidence interval = 0.07–0.954, *P* = 0.042) and resulted in significant improvements in the serum albumin level (mean increase = 16.3 g/l, *P* = 0.0003) and hemoglobin (mean increase = 2.68 g/dl, *P* = 0.0077). Long-term mTOR inhibitor treatment was well tolerated over an accumulated follow-up time of 29.8 patient years. No serious adverse events were reported. Early therapy with mTOR inhibitors offers effective, pathway-specific and personalized treatment for patients with JPI. Inhibition of the phosphoinositol-3-kinase–AKT–mTOR pathway mitigates the detrimental synergistic effects of combined *PTEN–BMPR1A* deletion. This is the first effective pharmacological treatment identified for a hamartomatous polyposis syndrome.

## Graphical Abstract

**Graphical Abstract:**
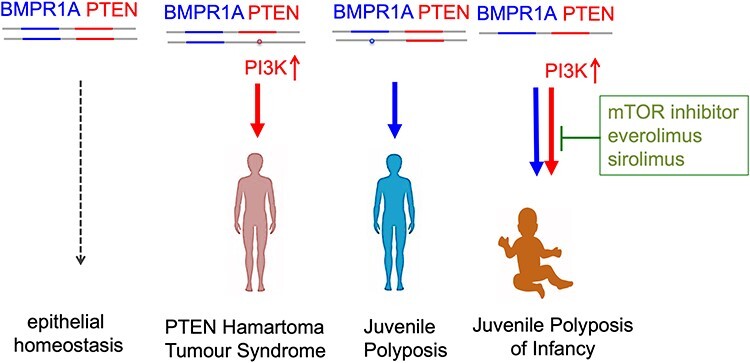

## Introduction

The study of rare genetic disorders can give insight into pathogenic mechanisms relevant to more common conditions, and targeting these mechanisms can provide proof of concept for personalized medicine. Dysregulation of signaling pathways controlling epithelial homeostasis is implicated in the pathogenesis of hereditary polyposis syndromes and sporadic colorectal cancer. Targeting those mechanisms and preventing tumor progression has been a research priority.

Phosphoinositol-3-kinase (PI3K)–AKT signaling and the bone morphogenetic protein (BMP) pathway control epithelial and mesenchymal crosstalk and maintain epithelial homeostasis. Hereditary hamartomatous polyposis syndromes result from dysregulation of these pathways. Germline loss of function mutations in phosphatase and tensin homolog deleted on chromosome 10 (*PTEN*), the master negative regulator of PI3K–AKT signaling, result in *PTEN* hamartoma tumor syndrome (PHTS) (1). Similarly, germline mutations of bone morphogenetic protein receptor type IA (*BMPR1A*), a key receptor in the BMP/SMAD signaling pathway, are a cause of juvenile polyposis syndrome (JPS).

PHTS encompasses individuals with Cowden syndrome and Bannayan–Riley–Ruvalcaba syndrome carrying germline *PTEN* loss of function mutations (1–3). PHTS is associated with a range of dermatological, neurological and vascular features and leads to increased cancer susceptibility (4–6). Patients with PHTS typically develop gastrointestinal symptoms secondary to polyp formation during young adulthood (1,7–9). Current recommendations are to initiate endoscopic surveillance for cancer at 35 years of age (10).

JPS is caused by germline defects in *BMPR1A* or *SMAD4* (11–14). Hamartomatous polyps typically develop in JPS by 14–20 years of age (11,15,16). Endoscopic surveillance is recommended starting at age 12 or earlier if there are gastrointestinal symptoms (14,17).

Since the *PTEN* and *BMPR1A* genes are located in close proximity on chromosome 10, *de novo* 10q23.3 microdeletions can result in haploinsufficiency of both genes. Combined loss of *PTEN* and *BMPR1A* has a synergistic effect, resulting in juvenile polyposis of infancy (JPI), a very severe hereditary polyposis syndrome. It presents in the first 2 years of life and is characterized by recurrent gastrointestinal bleeding, diarrhea, protein-losing enteropathy and intestinal obstruction secondary to polyp formation (18–20). It often requires early surgery and significantly reduces the lifespan of affected children. All published cases of *PTEN–BMPR1A* deletion syndrome have presented as sporadic cases, caused by a *de novo* microdeletion.

Polyposis syndromes are associated with a high risk of gastrointestinal and extraintestinal malignancy. Gastrointestinal symptoms, and the requirement for endoscopic surveillance and surgical management, result in significant morbidity. There are currently no established pharmacological treatment options for JPI or any of the hamartomatous polyposis syndromes (21,22). In familial adenomatous polyposis (FAP), chemoprevention strategies using combined cyclo-oxygenase and epidermal growth factor inhibitors show promise in clinical trials, but they are not in routine clinical use (22–24). Case reports have described clinical benefit from mammalian target of rapamycin (mTOR) inhibitors in JPI (25,26), PHTS (27) and FAP (23), but their efficacy has not previously been demonstrated in controlled studies.

This study summarizes phenotypic features of patients with JPI and shows that treatment of JPI patients with mTOR inhibitors reduces disease progression, reduces need for colectomy and corrects hypoalbuminemia and anemia as surrogate markers of epithelial barrier dysfunction and bleeding.

**Figure 1:**
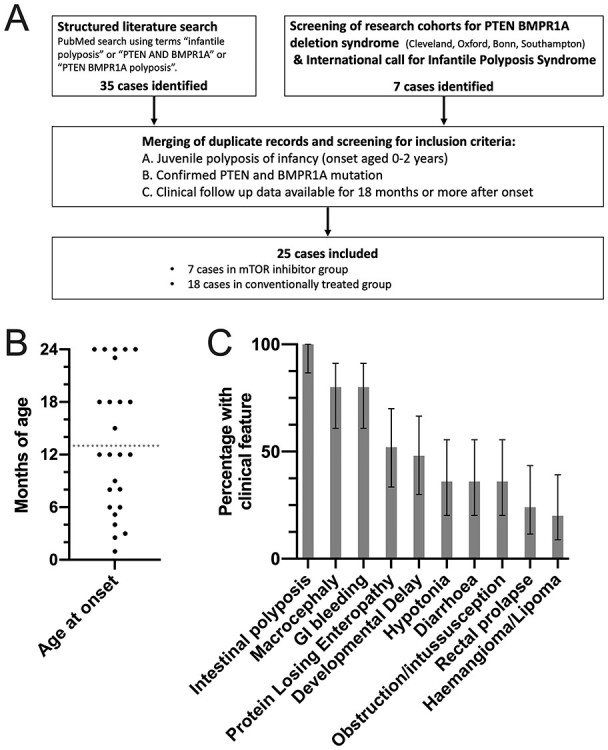
Case identification and characterization of JPI patients. (**A**) Strategies used to identify JPI patients with *PTEN* and *BMPR1A* deletions. (**B**) Age of onset of gastrointestinal symptoms for each included case (black dots). Patients presenting at 12 months of age or younger (below gray dotted line) had more severe phenotype. (**C**) Percentage of included cases with selected phenotypic features of PHTS and gastrointestinal complications (error bars show 95% confidence interval).

## Results

### Clinical features and natural history of JPI

A total of 25 patients with a definitive diagnosis of JPI resulting from confirmed microdeletions encompassing *PTEN* and *BMPR1A* genes were included in the study (Fig. 1A). All developed multiple gastrointestinal polyps at 0.08–2 years of age (Fig. 1B). Seven cases have not been previously reported (Supplementary Material, Table S1). The JPI patients presented with typical PHTS-associated features such as macrocephaly in 80% [95% confidence interval (CI) = 61–91], developmental delay in 48% (95% CI = 30–67), muscular hypotonia in 36% (95% CI = 20–55), hemangioma and/or lipoma in 20% (95% CI = 9–39) and penile freckling in 12% (95% CI = 4–30) (Fig. 1C).

Median age of onset of gastrointestinal symptoms was 1.0 year (range = 0.08–2.0 years). Symptoms included bleeding and anemia in 80% (95% CI = 61–91), protein-losing enteropathy or hypoalbuminemia in 52% (95% CI = 33–70), diarrhea in 36% (95% CI = 20–55), intestinal obstruction or intussusception in 36% (95% CI = 20–55) and rectal prolapse in 24% (95% CI = 11–43) (Fig. 1C).

Other than mTOR inhibitors, pharmacological treatment was limited to one patient who received a COX2 inhibitor (celecoxib) for 8 months. Blood transfusions, albumin infusions and iron supplementation were used as part of conventional treatment. Non-pharmacological interventions such as herbal and food supplements were not systematically reported.

Subtotal or total colectomy was performed in 60% of patients (mean age at colectomy = 3.2 ± 2.9 years, range = 0.83–10 years). Mortality rate was 16% (mean age at death = 4.2 ± 1.9 years; range = 3–7 years) and was from persistent rectal bleeding, nutritional failure and complications owing to surgery.

### Patients presenting aged 12 months or under have a more severe phenotype

Fourteen patients presented at 12 months of age or younger. These patients had a higher polyp burden than patients presenting between 1 and 2 years of age (RR of identifying >100 polyps = 6.46, 95% CI = 1.32–37.8, *P* = 0.015). This was associated with significantly greater risk of having protein-losing enteropathy (RR = 3.93, 95% CI = 1.34–14.32, *P* = 0.0082) or requiring colectomy (RR = 2.16, 95% CI = 1.02–5.34, *P* = 0.0325).

### mTOR inhibitor therapy was initiated in patients with a severe phenotype

Seven JPI patients received mTOR inhibitor treatment (mTOR inhibitor group), and 18 patients received standard of care without mTOR inhibitor (conventional treatment group). The median age of onset of gastrointestinal symptoms in the mTOR inhibitor group was 0.67 years (range = 0.42–1.50) compared with 1.38 years (range = 0.08–2.0) in the conventional treatment group. There was panenteric polyposis in both groups. Endoscopic images from previously unpublished cases in the mTOR inhibitor group demonstrate severe polyposis throughout the GI tract (Supplementary Material, Fig. S1). Prior to initiating therapy, all patients in the mTOR inhibitor group had anemia, and all but one had hypoalbuminemia.

### Treatment with mTOR inhibitors reduces risk of colectomy

**Figure 2:**
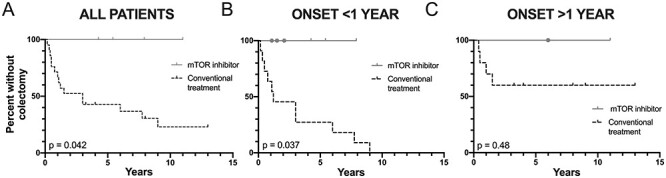
The effect of mTOR inhibitor therapy on time to colectomy in patients with JPI resulting from *PTEN* and *BMPR1A* deletion. Survival analyses showing time to colectomy from first onset of gastrointestinal symptoms in patients who received mTOR inhibitor (‘mTOR inhibitor’) and those who did not (‘conventional treatment’). Ticks indicate end point of patients who did not have colectomy. Dots indicate point mTOR inhibitor initiated. *P*-values calculated by Mantel Cox Log rank analysis. (**A**) All patients included. (**B**) Limited to patients presenting at 1 year of age or under. (**C**) Limited to patients presenting over 1 year of age.

Four patients initiated mTOR inhibitor therapy prior to colectomy. None of these patients have required colectomy during follow-up. The hazard ratio for colectomy was significantly lower in the mTOR inhibitor group than the conventional treatment group (hazard ratio = 0.27, 95% CI = 0.07–0.954, *P* = 0.042) (Fig. 2A).

Since disease severity and outcome differ depending on the age at presentation, we performed a stratified analysis. The colectomy rate among 11 conventional treatment patients presenting at age 12 months or under was 100% (median age of colectomy = 1.42 years, range = 0.83–10 years). Three patients in the mTOR inhibitor group presented in this age range (median age of commencing treatment = 1.50 years, range = 1.08–2.08 years), and none required colectomy (hazard ratio = 0.23, 95% CI = 0.056–0.91, *P* = 0.037) (Fig. 2B).

Among patients who presented at over 1 year of age, the conventional treatment patients had a colectomy rate of 36%, while the single patient who presented in this age range in the mTOR inhibitor group has not required colectomy (hazard ratio = 0.33, 95% CI = 0.014–7.8, *P* = 0.49) (Fig. 2C).

Four patients in the conventional treatment group died (mortality rate 22%) at a mean of 3.29 years after onset of gastrointestinal symptoms. None of the seven patients in the mTOR inhibitor group have died. There is no significant difference in survival distribution between groups (*P* = 0.26).

### Treatment with mTOR inhibitors improve protein-losing enteropathy and chronic gastrointestinal bleeding

**Figure 3:**
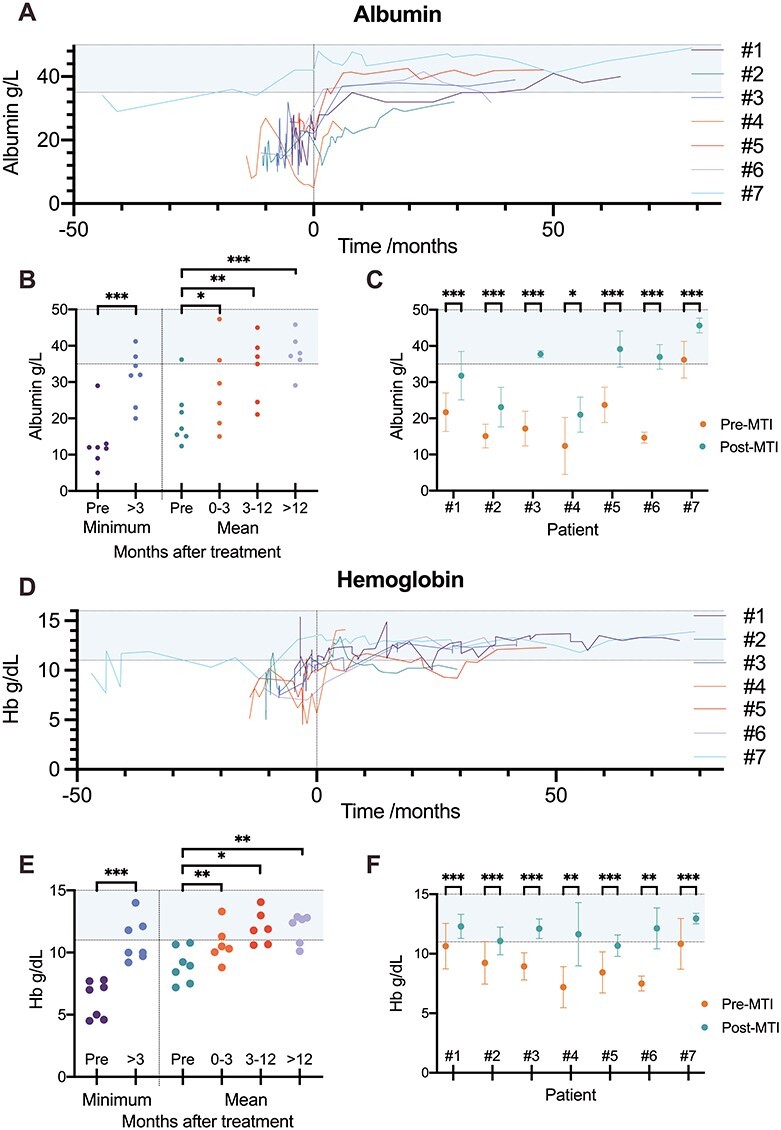
The effect of mTOR inhibitor therapy on hemoglobin and serum albumin concentration. (**A**, **D**) variation of serum albumin (A) or hemoglobin (D) concentration by time. Each participant initiated mTOR inhibitor therapy at *t* = 0. (**B**, **E**) A scatter plot comparing minimum serum albumin (B) and hemoglobin (E) concentration before and 3 months and onward on mTOR inhibitor treatment and comparing mean concentrations before and at three time points after initiation of mTOR inhibitor. Paired *T*-test performed to determine *P-*values. (**C**, **F**) Mean serum albumin (C) and hemoglobin (F) concentrations before and after initiating mTOR inhibitor therapy for each treated patient (error bars show standard deviation). Welch’s *T*-test performed to determine *P-*value. MTI = mTOR-inhibitor, # = patient number, ^*^P < 0.05, ^*^^*^P < 0.01, ^*^^*^^*^P < 0.001.

Hypoalbuminemia, resulting from protein-losing enteropathy, was observed in all patients in the mTOR inhibitor therapy group prior to initiating therapy (Fig. 3A). mTOR inhibitor therapy resulted in significant increases in mean serum albumin within 3 months of starting treatment, and these were sustained over the follow-up period (Fig. 3B). A significant increase in mean serum albumin was recorded in every treated patient (Fig. 3C). After 1 year of treatment, the mean increase in serum albumin concentration was 16.3 g/l (95% CI = 11.6–21.1, *P* = 0.0003).

Anemia, secondary to chronic gastrointestinal bleeding, was present in all patients in the mTOR inhibitor group before starting treatment (Fig. 3D). Mean hemoglobin concentration increased significantly following initiation of mTOR inhibitors (Fig. 3E). After 1 year of treatment, the mean increase in hemoglobin was 2.68 g/dl (95% CI = 1.08–4.28, *P* = 0.0077), and the increase in hemoglobin was significant in every patient (Fig. 3F).

After 3 months of treatment, the minimum serum albumin and hemoglobin concentrations did not fall below commonly used thresholds for correction (albumin concentration of 20 g/l and hemoglobin concentration of 8 g/dl) in any of the treated patients (Fig. 3B and E). The reduced requirement for blood transfusion and albumin infusion allowed the removal of a central venous catheter and facilitated discharge of one of the treated patients from hospital (Supplementary information: Clinical Narratives—Patient 4, Supplementary Material, Fig. S2).

### Effects of mTOR inhibitor treatment on health-care utilization, growth and development

**Figure 4:**
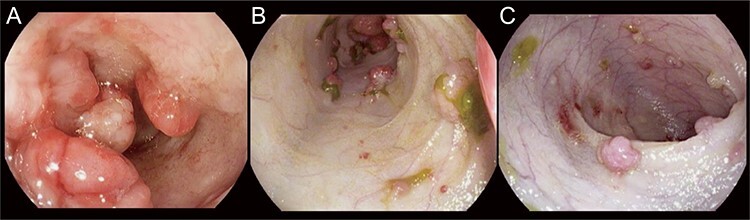
Endoscopic images showing decrease in polyp burden in patient with JPI treated with mTOR inhibitor. Endoscopic images of small intestine showing polyp progression from pre-sirolimus (**A**), 2 weeks of sirolimus (**B**) and 4 weeks of sirolimus (**C**).

Individual case reports of patients treated using mTOR inhibitors provide supportive evidence of their efficacy (Supplementary information: Clinical Narratives). A marked decrease in polyp burden following treatment was noted in patients 1–4 (Fig. 4), allowing the reduction in frequency of surveillance endoscopy.

Nutritional status was noted to improve in four patients. Patients 4 and 7 stopped total parenteral nutrition following initiation of treatment, and patients 4–7 gained several centiles in weight and height on a standardized growth chart.

Developmental delay is a feature of PHTS and is aggravated in patients with JPI by nutritional insufficiency and chronic illness. Patient 4 was noted to meet several new developmental milestones as their medical condition improved.

### mTOR inhibitors were well tolerated with no serious adverse events

The total cumulative time of mTOR inhibitor treatment was 29.8 years with 17.8 years cumulative follow-up on sirolimus and 12.0 years follow up on everolimus. Different dosing regimens were used, and the most common aimed for a trough sirolimus level of 5 ng/ml (Supplementary Material, Table S1). Both treatments were well tolerated. During the total of 29.8 years treatment, no serious adverse events have been noted (i.e. no hypertriglyceridemia, hypercholesterolemia or severe infections). Aphthous ulcers were noted in patient 1 on everolimus treatment. Treatment with mTOR inhibitors has been continued in all patients.

## Discussion

This study is the largest published cohort of cases with genetically confirmed JPI. It provides a summary of the phenotypic features, the severe clinical course and the pharmacological and surgical treatments used in this condition. It is the first study to compare a case series of patients managed with mTOR inhibitors against a control group and is the first to confirm that the therapeutic inhibition of the PI3K–AKT–mTOR signaling pathway has a significant clinical benefit in JPI.

The retrospective cohort analysis showed that mTOR inhibitors reduced the risk of colectomy in JPI. The reduction in colectomy risk was greatest in patients presenting aged 12 months or under, who had a more severe phenotype with aggressive polyp burden. Intraindividual quantitative analysis identified significant improvements in clinically relevant surrogate markers of gastrointestinal bleeding and protein losing enteropathy in all seven treated patients, and individual patient narratives identified other beneficial treatment effects. Everolimus and sirolimus were both well tolerated with no serious adverse events.

Clinical experience in ultra-rare genetic conditions is limited by the low number of patients worldwide and the lack of formalized trials to assess treatments. Different trial strategies are required to assess the treatment efficacy in these disorders. Our study is an observational, open-label and retrospective cohort study. It has several limitations: the number of patients who underwent treatment is small, the follow-up time per patient is limited and there are insufficient data to define the optimal choice or dosing schedule of mTOR inhibitor. However, the treatment effect observed is large, highly significant and transformative for patients. Publication bias was reduced by including all JPI patients who received mTOR inhibitors as well as all patients who have not as a comparator group. The international multicenter nature of the study reduced institutional bias and showed that the therapeutic effect was observed independently in seven centers.

This is the first controlled case series to demonstrate significant benefit from the pharmacological treatment of a hamartomatous polyposis syndrome and illustrates the potential benefits of mechanistically informed treatments in these conditions (22). Treatment benefit is clearer in JPI than in PHTS where everolimus and sirolimus have previously been used off-label (28). In PHTS, case reports describe the beneficial effects on vascular malformation (29), thymic hyperplasia (30) and cerebellar function scores (27), but significant effects on gastrointestinal polyp progression have not yet been demonstrated (31). These reports do demonstrate that long-term mTOR inhibitors are generally well tolerated, and the most frequent adverse events were mild-to-moderate headache, fatigue, hypercholesterolemia and mucositis (27–30). However, long-term side effects such as muscle wasting may be under-reported (32).

Translational research in ultra-rare genetic disorders offers exceptional insight into key pathogenic pathways and identifies potentially generalizable, targeted treatment strategies for more common disorders. This study highlights a therapeutic axis that may be relevant in the treatment of sporadic colon cancers, 20% of which have acquired 10q deletion (33).

**Figure 5:**
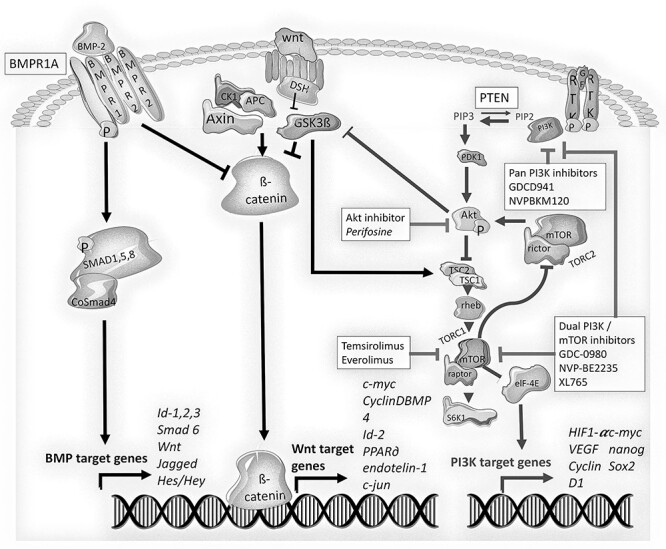
Signaling of the *PTEN*–PI3K and the *BMPR1A* pathway. A diagram of the PI3K and BMP/SMAD signaling pathways. BMP binding to BMPR complex causes *BMPR1A* activation and SMAD phosphorylation, nuclear translocation and transcription of SMAD target genes. *BMPR1A* signaling intersects with Wnt signaling by blocking ß-catenin activation and preventing Wnt target gene expression. *PTEN* controls the levels of PIP_3_, a key mediator of the PI3K–AKT–mTOR signaling pathway that regulates cell proliferation and survival. The complex intersection of signaling pathways at multiple levels of cell cycle control explains the synergistic defect caused by loss of *BMPR1A* and *PTEN* in chromosome 10 microdeletions. Therapeutic interference with this dysregulated signaling can be achieved via inhibition of several kinases that mediate the PI3K signaling pathway (including mTOR, AKT or PI3K inhibitors).

The BMP, *PTEN* and Wnt/ß-catenin pathways control the regeneration and development of the gut epithelium (34,35). They are dysregulated in hereditary polyposis syndromes and sporadic colorectal cancers. BMPs influence cell differentiation and tumorigenesis through their canonical role in SMAD signaling (Fig. 5) (36–38). BMP activation also blocks Wnt/ß-catenin signaling (39), and correspondingly, inhibition of BMP activates the Wnt/ß-catenin pathway in intestinal cells (36). Loss of *BMPR1A* reduces transcription of SMAD target genes and increases Wnt/ß-catenin signaling, which promotes cell proliferation. Similarly, loss of *PTEN* upregulates the PI3K–AKT–mTOR pathway, promoting cell proliferation and survival (Fig. 5) (40–42).

*PTEN–BMPR1A* deletion dysregulates both the BMP–SMAD and PI3K–AKT–mTOR signaling pathways. These pathways are largely independent, but there are relevant intersections which are informative when considering the efficacy of mTOR inhibitors. BMP activates *PTEN*, and BMP2 stabilizes and increases the expression of *PTEN* (43). *PTEN*, in reducing the activity of PI3K–AKT signaling, also decreases WNT signaling by reducing the AKT-mediated inhibition of GSK3ß and by preventing ß-catenin accumulation (36). mTOR inhibition is expected to partially correct disturbed PI3K signaling and re-establish some regulation of the Wnt signaling pathway, potentially explaining the significant clinical benefits observed in this study. More specific therapeutic targets that may be used in the future include PI3K inhibitors, dual inhibitors of mTOR and PI3K (40,41,44), or increasing *PTEN* expression via targeting the WW domain-containing ubiquitin E3 ligase 1-dependent degradation pathway (45) (Fig. 5).

There is currently neither a standard of care nor a pharmacological treatment for patients with JPI owing to *PTEN–BMPR1A* deletion syndrome (17). This study shows mTOR inhibitors can modify the severe clinical course of this condition and indicates that they should be considered as a primary treatment option, particularly in those presenting under 1 year of age or with a severe phenotype. A prospective multi-center study should be performed to confirm these findings. However, the observed treatment effect in this study questions whether a placebo-controlled trial of everolimus or sirolimus would be warranted.

## Materials and Methods

### Case identification and data collection

Previously unreported cases of JPI were identified by two strategies. First, genetic data from four large cohorts of PHTS and JPS patients were searched for chromosome 10 deletions encompassing *PTEN* and *BMPR1A* genes. The cohorts were compiled by specialist treatment centers in Oxford, Bonn, Cleveland and Southampton. Second, cases were identified by email survey on an international pediatric gastroenterology list server which has over 2700 subscribers, including pediatric gastroenterology fellows and consultants from at least 40 countries. Cases of infantile polyposis syndrome were requested and selected on a voluntary basis by the responding physicians. All participating institutions were advised to consult with their institutional review board prior to transfer of information. Data were obtained via a retrospective chart review by the responding physician and were anonymized prior to transfer via secure email. Reports were reviewed to identify and exclude duplicated patient information and to update follow-up data.

In addition, a structured search of the medical literature was performed to identify published reports of patients with JPI caused by *PTEN–BMPR1A* deletion syndrome. The PubMed database was searched using the terms ‘infantile polyposis’, ‘*PTEN BMPR1A*’, or ‘polyposis *PTEN BMPR1A*’. Results were limited to publications since 1997 (i.e. when *PTEN* was identified). References were screened to identify additional relevant reports.

For each patient, age at onset of gastrointestinal symptoms, age at diagnosis and last follow-up, presence of chromosomal deletion and extent, clinical features, gastrointestinal symptoms, number and distribution of polyps, gastrointestinal procedures and operations (whether a colectomy was performed and at what age) and whether patients received pharmacological treatment for the polyps were recorded. For patients who received treatment with an mTOR inhibitor, hemoglobin and serum albumin concentration values were collected. Responding physicians and authors of published reports were contacted to acquire the missing data and for additional follow-up data.

All patients in the final analysis fulfilled the following three inclusion criteria: (A) the presence of a confirmed germline deletion encompassing the *PTEN* and *BMPR1A* genes, (B) the presence of intestinal polyposis consistent with JPI with age of onset between 0 and 2 years and (C) clinical follow-up data available for at least 18 months after the onset of polyposis (Fig. 1).

### Retrospective cohort analysis

For the retrospective cohort analysis, outcomes were compared between patients who received treatment with everolimus or sirolimus in addition to normal treatment (‘mTOR inhibitor group’) and those who did not receive mTOR inhibitors but otherwise received standard of care (‘conventional treatment group’). Longitudinal clinical data and details of the treatment are reported for the mTOR inhibitor group. Serum albumin concentration and hemoglobin concentration were compared before and after treatment to assess individual outcomes, and surgical interventions including colectomy were recorded.

### Ethical considerations

For all previously unreported cases, patients or their guardians provided written informed consent for research as approved by the institutional review boards (Oxford GI biobank study and investigation of rare diseases sub-project; University of Oxford and University of Bonn). Each case met criteria for de-identified data in accordance with the HIPAA standards. The UT-Southwestern Human Research and Protection Program has determined that storage and analysis of de-identified data for this series does not constitute human subjects research as defined under federal regulations [45 CFR 46.102] and does not require institutional review board approval. All parameters investigated reflect the standard of clinical care.

### Reporting and statistics

Data were reported based on the STrengthening the Reporting of OBservational studies in Epidemiology (STROBE) guidelines (46). Statistical analysis was performed using GraphPad Prism (GraphPad Software, San Diego, USA). Statistical differences between groups were compared using Chi-squared test for dichotomous variables and Welch’s *T*-test for continuous variables. Changes in mean and minimum hemoglobin and serum albumin concentration were assessed using paired *T*-test. CIs were calculated by the Wilson/Brown method for proportions and by the Koopman asymptotic score for relative risks. Survival distributions were compared using the Mantel-Cox log-rank test. Signaling diagrams were created in PowerPoint (Microsoft, Redmond, USA) based on the graphical elements of Servier Medical Art (https://smart.servier.com/) and were edited using the GNU Image Manipulation Program (www.gimp.org/).

## Supplementary Material

PTEN_BMPR1A_JPI_supplementary_materials_ddab094
