# Supplementary material for: mTOR inhibitors reduce enteropathy, intestinal bleeding and colectomy rate in patients with juvenile polyposis of infancy with PTEN-BMPR1A deletion
Source: Hum Mol Genet. 2021 Apr 2;30(14):1273–82. doi: 10.1093/hmg/ddab094 (PMC8804886; doi:10.1093/hmg/ddab094)
Supplement: PTEN_BMPR1A_JPI_supplementary_materials_ddab094 [file pten_bmpr1a_jpi_supplementary_materials_ddab094.docx]

**mTOR inhibitors reduce enteropathy, intestinal bleeding and colectomy rate in juvenile polyposis of infancy due to PTEN BMPR1A deletion syndrome**

**Supplementary Materials**

**Contents Page**

**Supplementary Table 1: Details of included patients 2**

**Clinical Narratives for previously unpublished patients treated with mTOR inhibitors 4**

**Supplemental figure 1: Selected endoscopic images from previously unpublished cases**

**of JPI due to PTEN and BMPR1A deletion 8**

**Supplemental figure 2: Clinical course of a patient treated with mTOR inhibitor 9**

**References 10**

**Supplementary Table 1: Details of included patients**

| **Patient number** | **Deletion extent** | **Age of onset GI symptoms (years)** | **Colectomy** | **Treated with mTOR inhibitor** | **Authors** |
| --- | --- | --- | --- | --- | --- |
| 1  “Patient 1” | del BMPR1A Exons 6-13; del PTEN total | 0.75 | NO | Everolimus 0.3mg BD 0.55mg/kg/day | *Previously unpublished* |
| 2  “Patient 2” | Microdeletion within 10q23.2q23.31microdeletion involving PTEN and BMPR1A | 0.5 | NO | Sirolimus 0.04mg/kg/day | *Previously unpublished* |
| 3  “Patient 3*”* | del BMPR1A - PTEN total | 0.43 | YES* | Everolimus 3mg OD | *Previously unpublished* |
| 4  “Patient 4” | Microdeletion within 10q23.2q23.31 | 0.67 | YES* | Sirolimus 0.05mg/kg/day, aim trough 5-6 ng/mL | *Previously unpublished* |
| 5  “Patient 5” | del BMPR1A - PTEN total | 1.0 | NO | Sirolimus 4mg/day, aim trough 5ng/ml | *Previously unpublished* |
| 6  “Patient 6” | Microdeletion within 10q23.2q23.31 | 0.33 | YES* | Sirolimus 0.8mg/m^2^ BD, aim trough 5-8 ng/ml | *Busoni et al. 2019 [1]* |
| 7  “Patient 7” | del BMPR1A - PTEN total | 1.5 | NO | Sirolimus 0.055mg/kg/day, aim trough 4-5 ng/ml | *Quaranta et al. 2019 [2]* |
| 8 | 10q23.2-10q23.3 | 0.08 | YES | NO | Delnatte C et al. 2006 [3] |
| 9 | 10q23.2-10q23.3, | 0.21 | YES | NO | Delnatte C et al. 2006 [3] |
| 10 | 10q23.2-10q23.3 | 0.25 | YES | NO | Delnatte C et al. 2006 [3] |
| 11 | 10q23.2-10q23.31, | 0.5 | YES | NO | Babovic N et al. 2010 [4] |
| 12 | inv(10q)/del(10p) | 0.67 | YES | NO | Vargas Gonzales R et al. 2010 [5] |
| 13 | 10q22.3-10q24.1 | 1 | YES | NO | Jacoby RF et al. 1997 [6], Alimi A et al. 2015 [7] |
| 14 | 10q23.2-10q23.31 | 1 | YES | NO | Heald et al. 2010 [8] |
| 15 | 10q22.3-q23.32 | 1 | YES | NO | [Patrícia Horta Oliveira et al. 2013](https://www.sciencedirect.com/science/article/pii/S0022346812008925?via%3Dihub#!) [9] |
| 16 | 10q23 | 1.25 | YES | NO | Sweet K et al. 2005 [10] |
| 17 | 10q23.2-10q24.31, | 1.5 | YES | NO | Menko FH et al. 2008 [11] |
| 18 | 10q23.2–10q23.31 | 1.5 | YES | NO | Waisbourd-Zinman et al. 2016 [12] |
| 19 | PTEN and BMPR1A deletion | 1.5 | YES | NO | Arch EM et al. 1997 [13],Balciuniene J et al. 2007 [14] |
| 20 | *deletion of entire PTEN and most BMPR1A* | 1.92 | NO | NO | Vibede et al.. 2012 [15] |
| 21 | 10q23.2-10q23.31, | 2 | NO | NO | Menko FH et al. 2008 [11] |
| 22 | 10q23.2-10q.23.33 | 2 | NO | NO | Tsuchiya KD et al. 1998 [16] |
| 23 | PTEN and BMPR1A deletion | 2 | NO | NO | Heald et al. 2010 [8] |
| 24 | del BMPR1A Exon 9-13; del PTEN total | 2 | NO | NO | *Previously unpublished* |
| 25 | del BMPR1A - PTEN total | 2 | NO | NO | *Previously unpublished* |

*Colectomy performed prior to starting mTOR inhibitor

**Clinical Narratives for previously unpublished patients treated with mTOR inhibitors**

Patient 1 presented with JPI caused by a deletion of exon 6-13 of the BMPR1A gene and exon 1-9 of the PTEN gene (GRCh37 (hg19):g.(88,651,990_88,659,666)_(89,727,849_?)del), detected by multiplex ligation-dependent probe amplification (MLPA). The patient presented with macrocephaly (MRI revealed arachnoidal cysts), muscular hypotonia, blood in the stool and excessive sweating. At 6 months of age, a diagnosis of cow's milk protein allergy was suspected. Following recurrent episodes of intussusception, upper and lower endoscopy at 10 months of age revealed more than 50 juvenile polyps in the small bowel and colon. At one year of life, the patient underwent endoscopic removal of multiple colonic polyps and surgical removal of polyps in the small intestine. In view of the severity of the polyposis and anticipated need for multiple re-endoscopies, colectomy was considered. At the age of 13 months an mTOR inhibitor was started and never discontinued. Treatment commenced with Everolimus 0.3 mg, twice daily, which was subsequently increased to 1.5 mg twice daily. The patient is now 7.5 years old and has been free of abdominal pain.

Patient 2 had infantile-onset polyposis caused by chromosome 10 micro-deletion 10q23.2q23.31(88,626,815-90,254,694)x1 involving both BMPR1A and PTEN. From the age of 6 months, he presented with diarrhea and rectal bleeding. Stool frequency was 12 times per day with blood and mucus passed on every occasion. He was noted to have severe hypoalbuminemia and anemia. On endoscopic assessment, multiple juvenile polyps were identified throughout his gastrointestinal tract including the stomach, small bowel and colon. On repeat assessment over 100 polyps were noted to re-appear within a 3 - 4 month period. Laparoscopic-assisted endoscopic removal of small intestinal polyps was carried out on three occasions. In total, more than 500 polyps were removed. Sirolimus was started at 1.5 years of age. Since the Sirolimus treatment, progression of polyps has stopped and endoscopies have been required less frequently. The patient is currently 3.45 years old.

Patient 3 presented with JPI associated with a micro-deletion that spanned the entire BMPR1A and PTEN genes (GRCh37 (hg19):g.(?_88,516,426)_(89,727,849_?)del), detected by MLPA). The patient presented with hypotonia, macrocephaly, dilatation of the left brain ventricle and two arachnoid cysts at the age of 8 months. Gastrointestinal symptoms were noted from 5 months of age, and the patient developed a protein-losing enteropathy and recurrent intussusceptions. A definite diagnosis was made at 8 months of age. Endoscopies revealed numerous juvenile polyps in the stomach, duodenum and colon. At 9 months of age, a laparotomy was required due to acute obstruction caused by two small bowel intussusceptions. Colectomy was subsequently performed at 1 year of age. Due to ongoing symptoms caused by gastrointestinal polyps and protein losing enteropathy, Everolimus was started at age 1.3 years (dose of 3 mg per day, dosage stable since). Treatment with Everolimus reduced the frequency of new polyp development and slowed the growth of known polyps. No complications, such as intussusception or overt rectal bleeding, have been observed. The frequency of endoscopies has been reduced to surveillance once a year.

Patient 4 had macrocephaly and hypotonia. They presented at 8 months of life for “rectal prolapse” (passage of 2 cm of dark red tissue per rectum) and anemia. Flexible sigmoidoscopy revealed the presence of over 20 sessile and pedunculated polyps in the colon and rectum. Histopathology was consistent with juvenile polyps. Genetic testing confirmed a 1,056 kb microdeletion within Chr10q23.2q23.31 encompassing PTEN and BMPR1A. The patient re-presented at 10 months of life with recurrence of gargantuan polyp burden and he underwent repeat endoscopic removal of over 30 polyps from his rectum, colon, and small intestine. He was subsequently referred for and underwent colectomy. He continued to require repeated hospitalizations monthly for anemia and hypoalbuminemia for which central venous catheter was placed (Supplementary figure 1). He also had chronic intussusception involving 10-cm of small intestine. At 22 months of life, he had nadir albumin of 0.5 g/dL and hemoglobin 5.6 g/dL, at which time decision was made with family to begin Sirolimus 0.05 mg/kg. Improvement in symptoms, laboratory parameters, and growth were noted after 4 weeks of therapy. He was able to have central venous catheter removed. He continues to do well with trough level at 3-4 ng/mL. His hemoglobin has normalized, and he has not required further blood transfusions. Stool alpha-1-antitrypsin has normalized and albumin has stabilized. No complications have been observed on mTOR inhibition. The family is pleased with his improvement in energy and development.

Patient 5 had antenatal detection of macrocephaly, left ependymal cyst, and lumbar lipoma. They were born at full term with normal birth weight. At 12 months of age she presented with iron deficiency anemia. At 20 months of age she was referred to the pediatric gastroenterology service for diarrhea, anemia and hypoalbuminemia with intermittent rectal bleeding. Two months later the patient spontaneous expelled a polyp per rectum. An endoscopy revealed the presence of multiple hamartomatous polyps throughout the colon, stomach, duodenum and proximal jejunum. The patient had 5 episodes of intussusception at the age of 22 months, this required surgical reduction and surgically assisted small bowel polypectomy of more than 40 polyps. She required albumin and immunoglobulin infusions for protein losing enteropathy and iron infusions for anemia secondary to intermittent gastrointestinal bleeding. She underwent endoscopic polypectomy every 6-8 months with removal of more than 290 polyps. At the age of 25 months she was started on Sirolimus with improvement in Albumin levels. At the age of 4 years low grade dysplasia was detected. Currently she is 6 years 5 months old and has normal hemoglobin and albumin levels, with no rectal bleeding or diarrhea.

**Supplemental figure 1: Selected endoscopic images from previously unpublished cases of JPI due to PTEN and BMPR1A deletion**

**
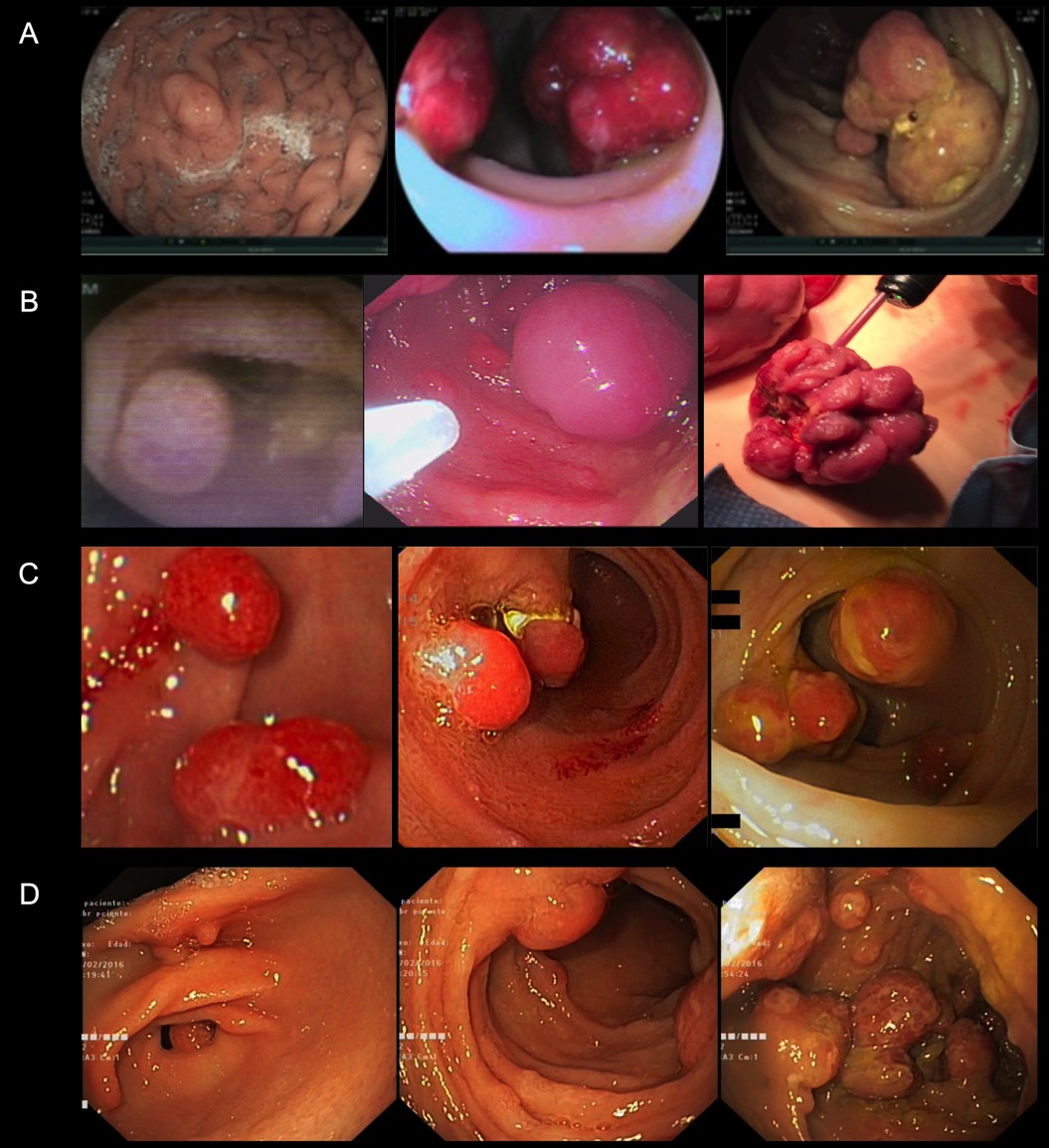
**

Selected endoscopic images from previously unpublished cases of JPI due to PTEN and BMPR1A deletion showing panenteric polyp distribution (**A**-**D**) All images described from left to right: **A** Patient 1. Endoscopy showing gastric, small bowel and colonic polyps. **B** Patient 2. Wireless capsule endoscopy image at 9 months showing small bowel polyp, colonic polyp, and excised polyp mass from laparoscopy-assisted enteroscopy. **C** Patient 3. Endoscopic images showing gastric, small bowel, and colonic polyps. **D** Patient 5. Endoscopic images showing gastric, small bowel, and colonic polyps.

**Supplemental figure 2: Clinical course of a patient treated with mTOR inhibitor**

**
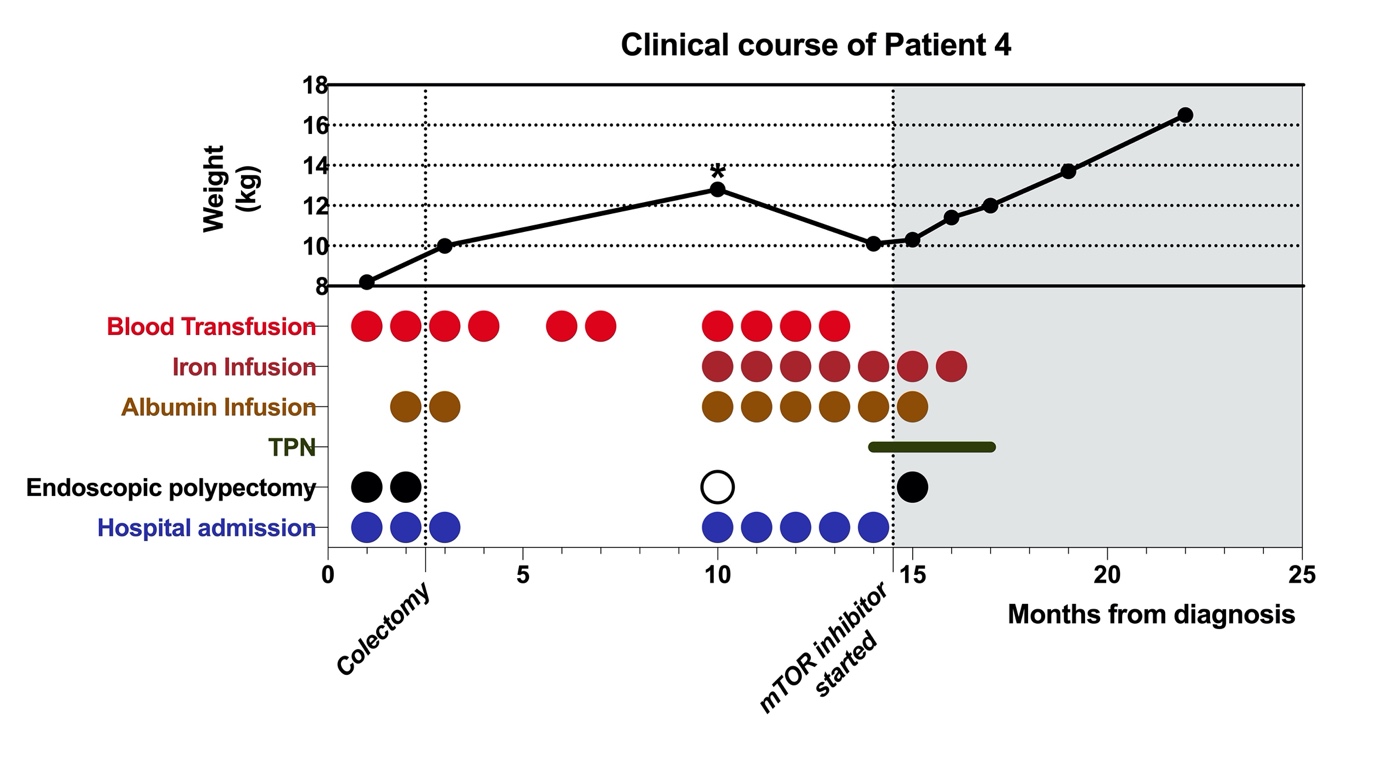
**

The clinical course of ‘patient 4’ is shown from the time of diagnosis through to the end of follow up. The time of colectomy and starting mTOR inhibitor are shown on the x axis, and the period of mTOR inhibitor treatment is shaded. Change in weight is shown in the top panel, and the timepoints of medical interventions are shown below. Filled circles represent completed interventions and empty circles attempted interventions. *At 10 months of life the patient was noted to be oedematous and fluid retention likely contributed to the increased weight.

**References**

1. Busoni, Veronica B., et al. "Successful Treatment of Juvenile Polyposis of Infancy With Sirolimus." Pediatrics 144.2 (2019): e20182922.
2. Quaranta, Muriel, et al. "Sustainable positive response to sirolimus in juvenile polyposis of infancy." Journal of pediatric gastroenterology and nutrition 68.2 (2019): e38-e40.
3. Delnatte C, Sanlaville D, Mougenot JF, Vermeesch JR, Houdayer C, Blois MC, et al. Contiguous gene deletion within chromosome arm 10q is associated with juvenile polyposis of infancy, reflecting cooperation between the BMPR1A and PTEN tumor- suppressor genes. Am J Hum Genet. 2006;78(6):1066-74.
4. Babovic N, Simmons PS, Moir C, Thorland EC, Scheithauer B, Gliem TJ, et al. Mucinous cystadenoma of ovary in a patient with juvenile polyposis due to 10q23 microdeletion: expansion of phenotype. Am J Med Genet A. 2010;152A(10):2623-7.
5. Vargas-González R, de la Torre-Mondragón L, Aparicio-Rodríguez JM, Paniagua- Morgan F, López-Hernández G, Garrido-Hernández MA, et al. Juvenile polyposis of infancy associated with paracentric inversion and deletion of chromosome 10 in a Hispanic patient: a case report. Pediatr Dev Pathol. 2010;13(6):486-91.
6. Jacoby RF, Schlack S, Sekhon G, Laxova R. Del(10)(q22.3q24.1) associated with juvenile polyposis. Am J Med Genet. 1997;70(4):361-4.
7. Alimi A, Weeth-Feinstein LA, Stettner A, Caldera F, Weiss JM. Overlap of Juvenile polyposis syndrome and Cowden syndrome due to de novo chromosome 10 deletion involving BMPR1A and PTEN: implications for treatment and surveillance. Am J Med Genet A. 2015;167(6):1305-8.
8. Heald B, Mester J, Rybicki L, Orloff MS, Burke CA, Eng C. Frequent gastrointestinal polyps and colorectal adenocarcinomas in a prospective series of PTEN mutation carriers. *Gastroenterology*. 2010;139(6):1927-1933.
9. Oliveira PH, Cunha C, Almeida S, Ferreira R, Maia S, Saraiva JM, et al. Juvenile polyposis of infancy in a child with deletion of BMPR1A and PTEN genes: surgical approach. J Pediatr Surg. 2013;48(1):e33-7.
10. Sweet K, Willis J, Zhou XP, Gallione C, Sawada T, Alhopuro P, et al. Molecular classification of patients with unexplained hamartomatous and hyperplastic polyposis. JAMA. 2005;294(19):2465-73.
11. Menko FH, Kneepkens CM, de Leeuw N, Peeters EA, Van Maldergem L, Kamsteeg EJ, et al. Variable phenotypes associated with 10q23 microdeletions involving the PTEN and BMPR1A genes. Clin Genet. 2008;74(2):145-54.
12. Waisbourd-Zinman O, Mamula P, Piccoli DA. Chromosome 10q23 Deletion Syndrome: An Overlap of Bannayan-Riley-Ruvalcaba Syndrome and Juvenile Polyposis Syndrome. J Paediatr Child Health. 2016;52(8):852. doi:10.1111/jpc.13289
13. Arch EM, Goodman BK, Van Wesep RA, Liaw D, Clarke K, Parsons R, et al. Deletion of PTEN in a patient with Bannayan-Riley-Ruvalcaba syndrome suggests allelism with Cowden disease. Am J Med Genet. 1997;71(4):489-93.
14. Balciuniene J, Feng N, Iyadurai K, Hirsch B, Charnas L, Bill BR, et al. Recurrent 10q22-q23 deletions: a genomic disorder on 10q associated with cognitive and behavioral abnormalities. Am J Hum Genet. 2007;80(5):938-47.
15. Vibede LD, Jensen UB, Sørensen TH, Pedersen LM. Bannayan-Riley-Ruvalcaba syndrome and juvenile polyposis in a two-year-old girl. Ugeskr Laeger. 2012;174(23):1614-5.
16. Tsuchiya KD, Wiesner G, Cassidy SB, Limwongse C, Boyle JT, Schwartz S. Deletion 10q23.2-q23.33 in a patient with gastrointestinal juvenile polyposis and other features of a Cowden-like syndrome. Genes Chromosomes Cancer. 1998;21(2):113-8.
